# A New Method for Classifying Scenes for Simultaneous Localization and Mapping Using the Boundary Object Function Descriptor on RGB-D Points

**DOI:** 10.3390/s23218836

**Published:** 2023-10-30

**Authors:** Victor Lomas-Barrie, Mario Suarez-Espinoza, Gerardo Hernandez-Chavez, Antonio Neme

**Affiliations:** 1 Instituto de Investigaciones en Matematicas Aplicadas y en Sistemas, Universidad Nacional Autonoma de Mexico, Mexico City 04510, Mexico; antonio.neme@iimas.unam.mx; 2 Facultad de Ingeniería, Universidad Nacional Autonoma de Mexico, Mexico City 04510, Mexico; mario.suarez@ingenieria.unam.edu; 3 Facultad de Ciencias, Universidad Nacional Autonoma de Mexico, Mexico City 04510, Mexico; lgerardohc@ciencias.unam.mx

**Keywords:** scene classification for SLAM, boundary object functions, RGB-D, bag of visual words, loop closing, SVM, 7-Scenes, SUNRGBD

## Abstract

Scene classification in autonomous navigation is a highly complex task due to variations, such as light conditions and dynamic objects, in the inspected scenes; it is also a challenge for small-factor computers to run modern and highly demanding algorithms. In this contribution, we introduce a novel method for classifying scenes in simultaneous localization and mapping (SLAM) using the boundary object function (BOF) descriptor on RGB-D points. Our method aims to reduce complexity with almost no performance cost. All the BOF-based descriptors from each object in a scene are combined to define the scene class. Instead of traditional image classification methods such as ORB or SIFT, we use the BOF descriptor to classify scenes. Through an RGB-D camera, we capture points and adjust them onto layers than are perpendicular to the camera plane. From each plane, we extract the boundaries of objects such as furniture, ceilings, walls, or doors. The extracted features compose a bag of visual words classified by a support vector machine. The proposed method achieves almost the same accuracy in scene classification as a SIFT-based algorithm and is 2.38× faster. The experimental results demonstrate the effectiveness of the proposed method in terms of accuracy and robustness for the 7-Scenes and SUNRGBD datasets.

## 1. Introduction

**Figure 1:**
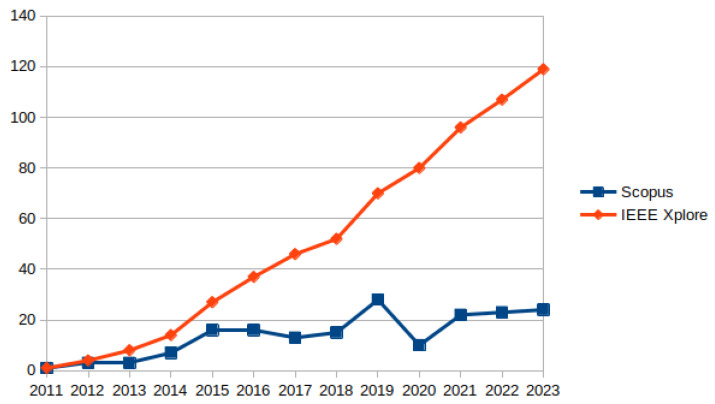
Number of research articles by year with the keywords: “RGB-D AND SLAM” in Scopus and IEEE Xplore from 2011 until August 2023.

Simultaneous localization and mapping (SLAM) is a critical problem in robotics and computer vision, which involves building a map of an unknown environment while simultaneously estimating the robot’s location within the map [1,2,3]. In recent years, RGB-D cameras have emerged as a popular sensing modality for SLAM systems, as they provide both color and depth information of the environment (Figure 1).

Scene classification in SLAM models that rely on the use of RGB-D cameras is a challenging task due to a number of factors [4,5]. Conventional image classification techniques like oriented FAST and rotated BRIEF (ORB) [6] and scale-invariant feature transform) [7] have been employed for scene classification within the SLAM context, utilizing only the 26 RGB channels. Yet, they do not consider depth. To address this problem, we propose a new method for scene classification in SLAM using the boundary object function (BOF) descriptor [8] on RGB-D points.

The BOF descriptor is a powerful technique for feature extraction and classification in computer vision. It converts the distance from the centroid to the points in the border of the object for each object found in a scene. The obtained distances are then used as the basis to classify the scene.

From an RGB-D camera, we extract points and fit them into orthogonal layers that are orthogonal to the camera plane. From each layer, we extract the boundaries of the detected objects, such as furniture, ceilings, walls, doors, etc. The extracted features are then classified using a machine learning method.

In this paper, we propose a new method for scene classification using the BOF descriptor on RGB-D points. Our method takes advantage of the RGB-D information provided by the camera and provides more robust and discriminative features for 3D scenes. We also use the concept of bag of visual words that are classified by an SVM, which allows us to handle complex scenes with high accuracy. Our experimental results demonstrate the effectiveness of the proposed method in terms of accuracy and robustness in different indoor scenes. We also provide experimental results to demonstrate the effectiveness of the proposed method in terms of accuracy and robustness in different indoor scenes.

The rest of the paper is organized as follows: Section 2 presents an overview of existing studies and contrasts them with the unique contributions of our research. Section 3 presents the proposed method in detail. Section 4 describes the experimental setup and presents the results. Finally, in Section 5, we present some conclusions and provide some directions for future work.

## 2. Related Work

Scene classification using RGB-D cameras is an active area of research in robotics and computer vision. In this section, we provide an overview of the related work in this field as well as a panoramic view of the state of the art.

Traditional image classification methods such as ORB or SIFT have been used for scene classification in SLAM systems. These methods rely on 2D image features and may not be sufficient for classifying 3D scenes accurately. In recent years, several methods have been proposed to address this problem [9].

A study of an RGB-D SLAM system for indoor dynamic environments used adaptive semantic segmentation tracking to improve localization accuracy and real-time performance, achieving a 90.57% accuracy increase over ORB-SLAM2 and creating a 3D semantic map for enhanced robot navigation [10].

Also, there is a pressing need to run scene or object detection algorithms on mobile objects such as robots and autonomous cars, where it is necessary to have lightweight algorithms that consume few computational resources (memory, processing time, and power). This is why algorithms based on that precept rescue simple feature extenders, as in [11]; the authors presented the modified R-ratio with the Viola–Jones classification method (MRVJCM) for efficient video retrieval, achieving 98% accuracy by automating image query recognition and optimizing system memory usage.

The BOF descriptor has been widely applied in several contexts. It was introduced in [8], where the descriptors allowed an accurate recognition of assembly pieces, including several shapes such as squares and circles; at the time, the orientation was determined by the shadow that the pieces projected. The images from which the BOF descriptors were obtained were taken from a camera located at the top of an assembly facility, which facilitated the detection of objects. A neural network, fuzzy ARTMAP, conducted the classification stage of the pieces, and the results were highly precise for all combinations. In a more recent application [12], it was applied in a technique to identify objects from several viewing perspectives. A condensed convolutional neural network model, inspired by LENET-5, was employed for the classification phase. This approach was implemented on an FPGA.

The BOF consists of a numeric vector used to describe the shape of an object. It differs from local feature extraction descriptors like SIFT, SURF, and ORB in that it describes the shape of an object but not the neighborhood of a feature point.

The steps to obtain a BOF descriptor are as follows:

1. Apply an object segmentation procedure.
2. Detect the contour and centroid of the object.
3. Quantize the contour into *n* points, where *n* is the size of the descriptor. With n=180, the test guarantees a good balance between accuracy and computer performance [13].
4. Obtain the distances from the quantized contour to the centroid.
5. Concatenate the distances in counterclockwise order of appearance.
6. Normalize the vector (the components are divided between the maximum components).

In recent years, the application of neural networks, in particular those with a deep learning architecture, in the field of scene classification has witnessed a significant increase. Heikel and Espinosa-Leal [14] implemented a YOLO-based object detector that gives a descriptor of each image this was put in Tf-idf representation; finally, the information was classified using random forest. The pipeline is similar to ours, with the difference being that we use a support vector machine for classification and BOF as the descriptor. Another deep learning approach is an autonomous trajectory planning method for robots to clean surfaces using RGB-D semantic segmentation, particularly employing the double attention fusion net (DAFNet), presented in [15]. This technique enhances indoor object segmentation and, through various processes, generates a smooth and continuous trajectory for the robotic arm, proving effective in surface cleaning tasks.

In Ref. [16], the authors combined deep learning and RGB-D sequences to take advantage of all the RGB-D information provided by Kinect. Their efforts included fussing the color and depth information with three techniques, namely, early, mid, and late fusion. A ConvNet-based method was used to extract descriptors due to the capacity of generalization that this type of structure allows. The results were significantly better in indoor scenarios than those obtained by the bag of visual words (BOVW) approach. The main drawback of the this ConvNet-based system is linked to the difficulty of its implementation in real-time situations due to the its high demand for computing power.

Semantic information is an important feature in interactive robot assistants. In Yuan et al. [17], the authors took advantage of the semantic segmentation provided by the Panoptic feature pyramid networks. This incorporation allows the system to create a semantic codebook, which divides the words in dynamic and static tokens. The rationale behind this approach is that the static words are more meaningful, whereas the dynamic ones have less value. For example, the word *person* has a value of zero because people cannot describe a place. Their descriptor is built upon a semantic graph, which also serves to define a similarity function.

Finally, a model in which the use of residual neural networks to optimize traffic sensor placement and a subsequent predict of the network-wide origin-to-destination flows is presented in [18]. The proposed deep learning model offers high prediction accuracy, relying on fewer sensors, as demonstrated on the Sioux Falls network.

## 3. Materials and Methods

In this section, we describe the materials and methods used in our proposed method for scene classification in SLAM using the BOF descriptor on RGB-D points.

### 3.1. Dataset and Platform

We based our experiments on three datasets (Table 1) for the training and testing stages: the Microsoft 7-Scenes [19], SUN RGB-D, and OfficeBot TourPath (OBTP) datasets, adhering to the train–test split as prescribed in the original publication [20]. The three datasets furnish color and depth information about the environment, a crucial requirement for our proposed method.

The results were procured using an Jetson Nano single-board computer (NVIDIA Corporation, Santa Clara, CA, USA) running on Ubuntu 18.04.6 LTS. The system specifications include a CPU clocked at 1.479 GHz and 4 GB RAM.

### 3.2. BOF Feature Extraction from RGB-D Images

In this method, we use only depth images to extract BOF features by following these steps:

1. The depth image is transformed into a point cloud, which is a set of 3D points representing the position of the objects in space captured by the image.
2. The point cloud is divided into layers. The number of layers is a hyperparameter *L* that is set before extracting the BOF features. We select an axis determined by a unitary vector *v* and project the points to *v*.
  (1) $$proj_{v}\left(p\right)=p\cdot v$$
  After that, we obtain the minimum $\min_{v}$ and the maximum $\max_{v}$ of these projections and divide the interval $\left[\min_{v},\max_{v}\right]$ into *L* subintervals of length $l=\frac{L-1}{\max_{v}-\min_{v}}$. Finally, using the function ⌊x⌉, which rounds a float to an integer, an index I(p) is assigned to each point *p* by the following equation:
  (2) $$I\left(p\right)=\lfloor l\;\left(proj_{v}\left(p\right)-\min_{v}\right)\rceil$$
  All points contained within a layer are projected to a plane perpendicular to the roll axis of the camera. In this manner, points are represented in the form of (x,y) for further analysis.
3. For each layer obtained in the previous step, a binary image of resolution W×H is generated, consisting of ones in the grids containing at least one point in space and zeros where there is no point. To determine if the pixel of the new binary image with index i,j is 0 or 1, we use an index function I(x,y) that assigns the two-dimensional integer formed by two coordinates to each projected point (x,y) in a layer, which results form the rounding function ⌊x⌉ according to:
  (3) $$I\left(x,y\right)=(\left\lfloor l_{x}\;\left(x-\min_{x}\right)\right\rceil ,\left\lfloor l_{y}\;\left(y-\min_{y}\right)\right\rceil )$$
  where $\min_{x}$ and $\min_{y}$ are the minimums of the projections in the canonical axes *x* and *y*, $l_{x}=\frac{W-1}{\max_{x}-\min_{x}}$ and $l_{y}=\frac{H-1}{\max_{y}-\min_{y}}$. Once the index I(x,y) is determined, the binary image is constructed following the next rule: given a pixel of the binary image (i,j), if there exists $\left(x_{0},y_{0}\right)$ such that $I\left(x_{0},y_{0}\right)=\left(i,j\right)$, we set the value of the pixel (i,j) to 1; otherwise, the value of the pixel (i,j) is set to 0.
4. The binary image is smoothed to eliminate the gaps caused by the low resolution of the point cloud. Smoothing is achieved using a closure morphological operation.
5. For each binary image, closed contours are found.
6. For each contour, the BOF descriptor is extracted following the steps discussed in Section 2.
7. All extracted BOF descriptors are stacked and associated to the frame.

**Figure 2:**
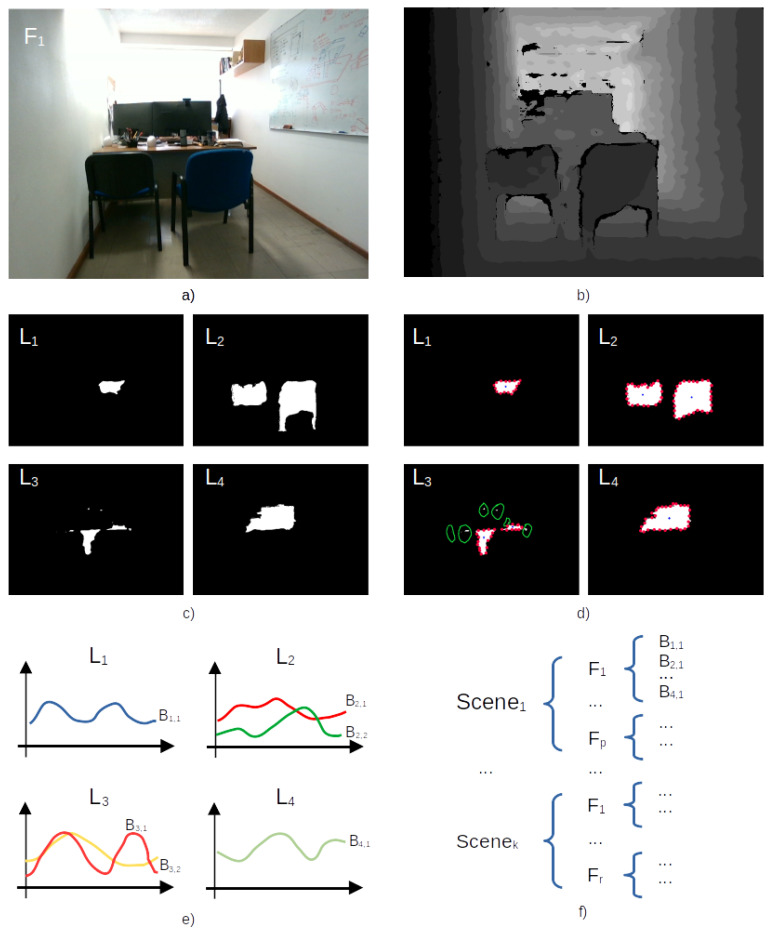
BOF feature extraction process. (**a**) RGB image, (**b**) depth image, (**c**) binary images representing various layers at different depths, and (**d**) object BOF (in red) and centroid (in blue) from several layers, (**e**) BOF descriptors per layer, and (**f**) BOF descriptors stacked and associated to the frame. Contours with at least 1% of the total area are indicated by green highlighting.

Figure 2 illustrates the aforementioned process. It is important to note that only the depth image is take in into account, and the RGB image is kept aside. In Figure 2c, the multiple layers display objects highlighted with 1’s. A filter smooths the binary images to minimize noise. In Figure 2d, the Boundary Object Function is extracted solely from objects where the contour comprises a minimum of 1% of the total area.

### 3.3. Scene Classification

As a complement to autonomous navigation, scene recognition [22] endows an intelligent system with the ability to localize itself and understand the context of its surroundings. By recognizing the place where it is located, the intelligent system can adapt its actions to achieve its goals, e.g., for the case of a mobile robot, to move from one point to another or to plan based on location-derived information.

**Figure 3:**
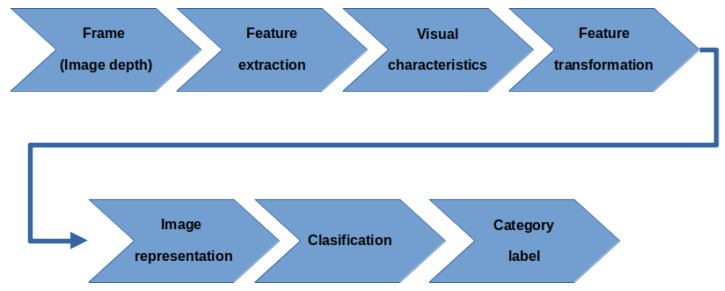
General scheme for image classification.

For this purpose, a scene recognition system based on traditional methodologies is proposed. This scheme is presented in Figure 3.

For the feature extraction stage, the traditional methodologies include algorithms such as SIFT, SURF, and ORB. In the feature transformation stage, BoVW approaches are commonly applied. For the classification stage, models such as support vector machine (SVM), random Fforest, naïve Bayes, or k-nearest neighbors (kNN) are commonly applied.

The contribution of this work involves following the BOF perspective as a feature extraction method. The reason for this is the relatively low computational demand required for obtaining of this descriptor compared with that of other commonly used local feature extraction schemes, such as the mentioned SIFT, SURF, and ORB methodologies.

SLAM algorithms need a loop closure mechanisms to ensure the correct generation of the map, detecting revisited places in order to add consistency and robustness. When the main sensor of the robot is a camera, it is referred to as appearance-based loop closure detection. In [23], these mechanisms belong to two categories, namely, offline and online. The former, to which our BOW approach belongs, needs a dictionary or database with information trained previously. Bag of binary words [24] is one of the most important exponents of the offline type. It was used, for example, in ORB-SLAM [25] and has been tested more recently in [26].

Given a training set of BOF descriptors, a codebook needs to be created. The codebook is an array of centroids $c_{i}$. To represent a BOF descriptor $\left(B_{1},\cdots ,B_{n}\right)$ as a word, we calculate the distance of each component $B_{j}$ with each centroid $c_{i}$ and select the closest. So, the vector $\left(c_{i}^{1},\cdots ,c_{i}^{n}\right)$ is formed. Finally, the number $f_{i}$ counts the times that the centroid $c_{i}$ appears in $\left(c_{i}^{1},\cdots ,c_{i}^{n}\right)$; the result is a *k* length vector $\left(f_{1},\cdots ,f_{k}\right)$, which represents the frequency that each word $c_{i}$ has in the BOF descriptor. All this process is summarized in the map:

(4) $$\left(B_{1},\cdots ,B_{n}\right)\mapsto \left(f_{1},\cdots ,f_{k}\right)$$

### 3.4. Loop-Closing Detection

We followed the method described in [27] to perform loop closing, under two constraints: first, we assume that the point clouds of visited frames are already stored; second, we use a simple bag of words dictionary without a tree structure. In other words, we apply k-means and not hierarchical k-means for its creation in order to keep computational complexity as low as possible.

The BOW descriptor obtained with Equation (4) needs to be described in Tf-idf representation with the following map:

(5) $$\left(f_{1},\cdots ,f_{k}\right)\mapsto \frac{1}{\sum_{1}^{k}f_{i}}\left(f_{1},\cdots ,f_{n}\right)\cdot \left(w_{1},\cdots ,w_{n}\right)$$

The vector of weights $\left(w_{1},\cdots ,w_{n}\right)$ is obtained in the training phase by:

(6) $$w_{i}=\log\left(\frac{|X_{train}|}{\nu_{i}+1}\right)$$

where $|X_{train}|$ is the number of BOF descriptors in the training set, and $\nu_{i}$ counts those that contain the word $c_{i}$.

The applied distance in the whole process is the $L_{1}$-norm. The justification of relying on this metric comes from the results reported in [28], where it outperformed normalization. The BOW vector associated to the frames *i* and *N* are compared using the function:

(7) $$s\left(i,N\right)=1-\frac{1}{2}\left|\left|\frac{v_{i}}{\left|\right|v_{i}\left|\right|}-\frac{v_{N}}{\left|\right|v_{N}\left|\right|}\right|\right|$$

where *N* represents the label of the current frame. In order to normalize this function and given that the object of study is sequences of images, the following variation is used as a similitude score:

(8) $$\eta \left(i,N\right)=\frac{s(i,N)}{s(N-\gamma ,N)}$$

where γ is an integer interval such that the frame N−γ passes one second before the current frame *N*.

If s(N−γ,N) is less than 0.1, the frame is discarded; otherwise, the frame $i^{*}$ that maximizes η(i,N) is inspected. A time consistency check is carried out for this maximum, which consists of the replication of these steps for frames $N-T_{1},N-T_{2},\cdots ,N-T_{m}$, validating that the corresponding maxima $i^{*},i_{1}^{*},\cdots i_{m}^{*}$ are indeed closed enough. Two thresholds $\alpha^{+}$ and $\alpha^{-}$ are selected. If $\eta \left(i,N\right)<\alpha^{-}$, the frame is discarded. If $\eta \left(i,N\right)>\alpha^{+}$, the frame is accepted as a loop-closing one. However, if η(i,N) is in the range ($\alpha^{+}$, $\alpha^{-}$), a geometric verification using RANSAC over the point clouds corresponding to the frames *i* and *N* is needed.

### 3.5. Experimental Setup

We conducted experiments on a dataset of indoor scenes captured using an RGB-D camera. The dataset contains several scenes with different illumination conditions as well as distinct object configurations. We compared the performance of our proposed method with that of traditional image classification methods such as SIFT and GIST [29].

In the context of scene classification, we trained two models: the first one relies on BOF for the feature extraction stage, whereas the second is based on SIFT. Both models use BoVW and SVM for feature transformation and classification, respectively. For the purpose of this paper, we call the first method *BOF-BoVW* and the second *SIFT-BoVW*.

For the experiments, we used the Microsoft 7-Scenes dataset [19], which consists of RGB-D sequences (recordings) in 7 different zones. Each zone has different sequences. The zones are Chess, Fire, Heads, Office, Pumpkin, RedKitchen, and Stairs.

**Figure 4:**
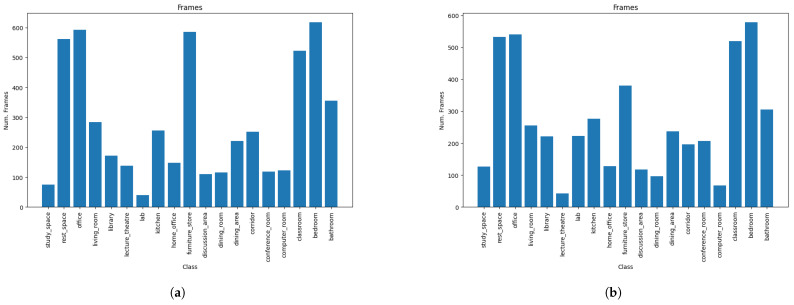
Train and test number of frames per scenes for the SUN RGB-D dataset. The scenes labels, from left to right, are study space, rest space, office, living room, library, lecture theatre, lab, kitchen, home office, furniture store, discussion area, dining room, dining area, corridor, conference room, computer room, classroom, bedroom, and bathroom. (**a**) Train split with 5285 frames. (**b**) Test split with 5550 frames.

Also, we performed tests sing the SUN RGB-D dataset with the same train–test split as in the original publication [20]. The dataset consists of several thousands of images distributed along 19 labeled scenes; the split was chosen carefully by the authors in order to avoid the sparsity of the frames and allow a correct generalization (Figure 4). Originally this dataset was tested using a GIST descriptor linked to a SVM. The stack of the GIST descriptors applied to RGB and depth improved the results. The best results were achieved with the use of the Places-CNN descriptor and an RBF-SVM.

We were interested in comparing our model using this dataset because it is based on an an SVM approach. This provided a direct metric to compare our results with the existing ones.

In order to prove the effectiveness of the scene classification in real conditions, we tested the BoVW-BOF method with our own robot platform, which has a camera (RGB-D realsense model D45)5. For the training phase, we recorded 7 scenes in our laboratory: office_1, office_2, laboratory_1, corridor_1, corridor_2, corridor_3, and bathrooms. We recorded the depth and RGB images and collected them to create the OfficeBot TourPath (OBTP) dataset.

For the loop detection experiments, we concentrated on the chess sequences in the Microsoft 7-Scenes dataset. We followed the split for the training and testing sets as described in [30]. For the training set, we created a code book of 1024 words based on the BOFs descriptor extracted from the sequences; for testing, we used the third sequence. Then, we put each word in a TF-IDF representation and compared the similarity of the current frame with the one *N* frames behind, as stated in Section 3.4. After temporary verification, we fixed the thresholds α and $\alpha^{-}$ as in [27] in order to determine if a loop candidate is approved or discarded.

In the next list, we describe the parameters that modulate the behavior of the algorithm:

1. $\alpha^{+}$: Upper threshold that allows us to determine if a loop is accepted.
2. $\alpha^{+}$: Lower threshold that allows us to determine if a loop is discarded.
3. *N*: If the current keyframe is in position *M*, then the keyframe M−N is used to calculate the normalization factor η(M,M−N).
4. $\tau_{N}$: The threshold that the normalizer has to exceed in order to be accepted.
5. TC req: Number of keyframes adjacent to the current frame that are required to declare it as valid in the temporary consistency check.
6. TC: Number of keyframes in which the temporary consistency check runs.
7. $\tau_{TC}$: Threshold that represents the maximum difference allowed between the index $i^{*},i_{1}^{*},\cdots ,i_{M}^{*}$ that maximizes the normalized scores η of the frames adjacent to the current one.
8. keyframes: The number of frames that are considered in evaluation. It is the result of a homogeneous division of the number of total frames.

The next list contains the values returned as output by the algorithm [

1. Candidates: Number of keyframes that pass the upper $\alpha^{+}$ threshold.
2. Approved: Number of candidates that pass the time consistency check.
3. Discarded: Number of keyframes that stay below the $\alpha^{-}$ threshold.

## 4. Results

### 4.1. Results for Scene Classification on Microsoft 7-Scenes Datasets

We first evaluated *BOF-BoVW* and *SIFT-BoVW* using the hold-out method, with 75% training data and 25% test data, from a single sequence per class.

**Figure 5:**
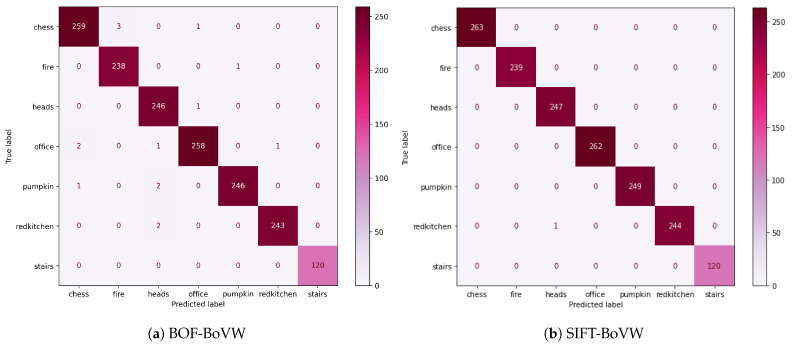
Confusion matrix for BOF-BoVW (**a**) and SIFT-BoVW (**b**) using hold-out method with 25% test data.

In the classification stage and using cross-validation, we found that the optimal classifier parameters are C=3.58 with an RBF kernel for *BOF-BoVW* and C=0.01 with a linear kernel for *BOF-BoVW*. Figure 5 shows the confusion matrices resulting for the parameters mentioned. Table 2 shows that we observed an accuracy of 99% with our proposed method, almost reaching the accuracy of SIFT-BoVW, which has just one mismatching frame. This scenario has applications for a robot that navigates in the same building.

**Figure 6:**
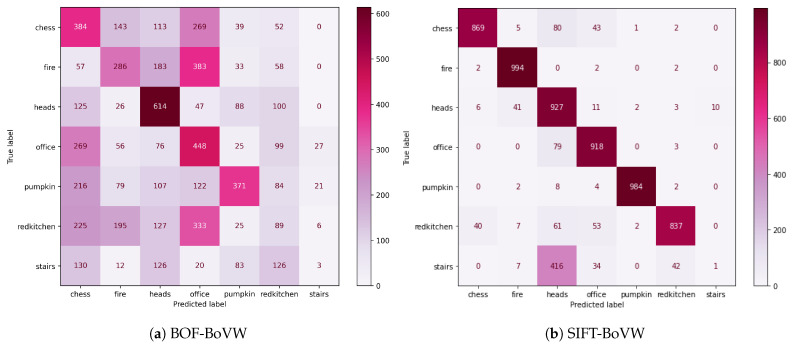
Confusion matrix for BOF-BoVW (**a**) and SIFT-BoVW (**b**) using a different sequence as test data.

In the next stage, *BOF-BoVW* was evaluated using a sequence of frames different from the ones present in the training set as testing data. This scenario is applicable to robots that navigate in unknown buildings. In Figure 6, we show that our method decays to 34% accuracy, where the heads scene is the one with the best performance metrics. It can be observed that the three blocks in the central diagonal of Figure 6a are consistent. Conversely, SIFT-BoVW maintains high accuracy, where the decrease is justified by the unbalanced stairs class. From this, the diagonal in Figure 6b only fails in the last square. The Table 3 shows an accuracy of 34% for *BOF-BoVW* and 85% for *SIFT-BoVW*.

### 4.2. Results for the SUN RGB-D Dataset

The SUN RGB-D dataset allows testing the generalization capabilities of SVM models. To achieve the best results with the BOF descriptor, we set the number of layers to 20 in the point cloud. Each layer produces a binary image of 300 × 300 pixels, from which we obtain the contours. We requested that the area of the contour was at least one percent of the total binary image area. With this configuration, 164,972 vectors were obtained, leading to 5285 BOF descriptors, one for each frame of the training set.

**Figure 7:**
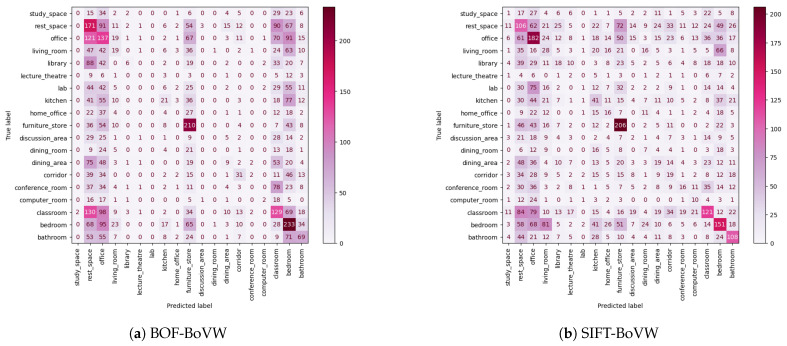
Confusion matrices of the test split for the SUN RGB-D dataset.

The SUN RGB-D dataset is known for presenting several challenges, a fact that is confirmed by the confusion matrices displayed in Figure 7. It can be observed that the matrices are disperse, and just some squares of the diagonal are colored, indicating the difficulty of achieving low error. Along this line, the class of furniture store objects is the one with higgest F1 score. In Table 4, we show that in some scenes, such as some from the study space class, the SIFT-BoVW model achieves better results, whereas in others classes, such as the rest space one, the BOF-BoVW model obtains the best results. In terms of expected accuracy, both methods offer similar results.

Originally, in [20], the SUNRGB dataset was evaluated with a configuration of the GIST descriptor and an SVM as the classifier. In addition, the color and deep information were included in the evaluation. In Table 5, we compare our implementations with the traditional approaches. Of particular interest is the observation that BOF-BovW performs better than GIST with either RGB or depth information alone. From this, we conjecture that the use of both color and depth information is needed to improve the GIST performance.

The deep analysis of the performance of our model was based on the impact of the number of BOF descriptors per frame. We varied it from three to twenty in order to examine the changes in the classification metrics.

### 4.3. Results for Real Usage Conditions

**Figure 8:**
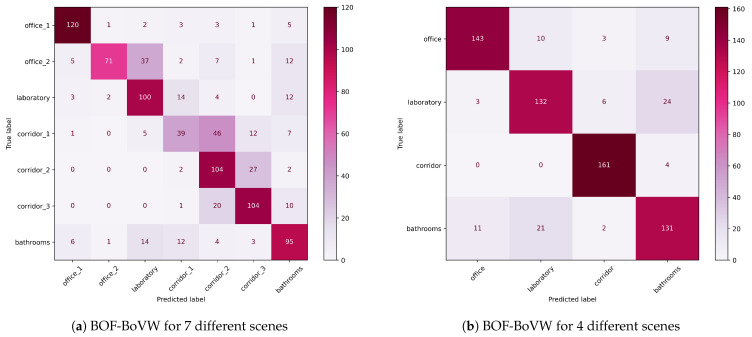
Confusion matrices of the test split on the OBTP dataset.

We tested the the BoVW-BOF approach with our mobile robot platform; we built our own OBTP dataset (Table 1). For the training phase, we considered seven scenes; a total of 31,000 BOF descriptors were extracted from 1570 depth images. In the testing phase, the robot was launched on a different day with the same illumination conditions, and 920 frames were evaluated. Figure 8 shows two different confusion matrices. We noticed that the corridors were similar scenes in terms of the absence of characteristic objects. Also, the office_2 scenes had less training frames than the rest. So, in Figure 8b, we restrict our scenes to the those determinants resulting in an improvement in accuracy of up to 86% (Table 6).

**Figure 9:**
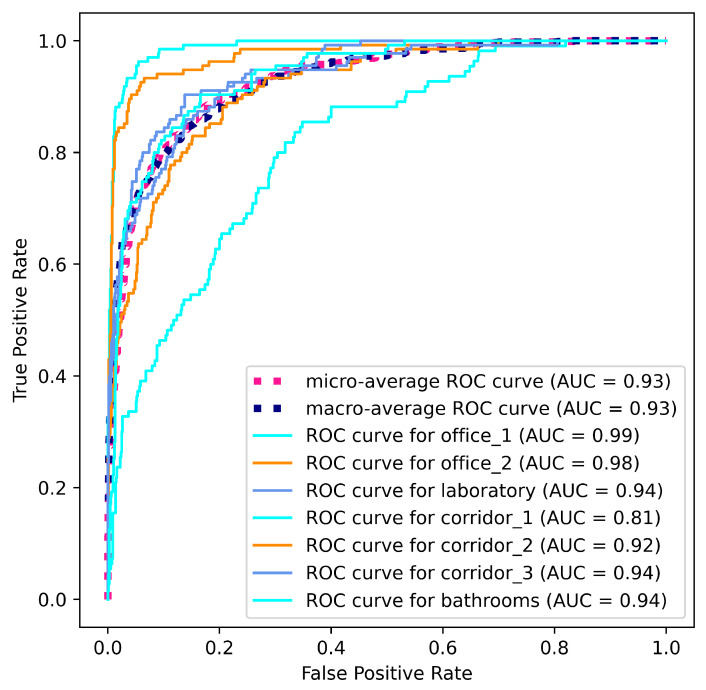
Receiver Operating Characteristic curve on the OBTP-DS.

In order to check the efficiency and performance of the described method, an ROC curve was generated (Figure 9) on the OBTP dataset. It can be observed that most of the scenes are satisfactorily classified, except for the corridor_1 scene. The main reason for this discrepancy is the significant imbalance in the number of frames in that scene compared to the remaining ones. For the latter scenes, the area under the curve (AUC) is above 0.92.

### 4.4. Results for Time Performance

The main objective of using BOF over SIFT is to reduce the computational complexity associated with the whole process, which includes memory (hardware) and processing time, to enable real-time recognition on single-board computers. To compare the consumption of computational resources, a comparison is made between the use of BOF and SIFT descriptors.

Our results are presented from two aspects: CPU usage time and a stage that we call “real time”. The CPU time combines user and kernel times and accounts for each core in multi-core processors. The real-time aspect refers to the total elapsed time from the start to the end of the process, not considering individual core times. In multi-core processors, these measurements can differ, especially if processes run in parallel, which may influence the actual time in order to make it shorter than the CPU time.

The processes evaluated in Table 7 are

1. Extraction of descriptors from a frame, which is the average value obtained from 10 runs on the same frame is considered as the relevant quantity.
2. Extraction of descriptors from multiple frames, where 1000 frames were processed.
3. Generation of a visual word vocabulary, consisting of 1024 words. For BOF-BoVW, a three-layer case was computed on 34,000 samples. BOF-BoVW 20-layer case was computed over 190,000 samples, and the SIFT-BoVW case was computed on 150,000 samples.
4. Further transformation to a BoVW TF-EDF representation using the 1024 words dictionary.
5. Training of the model using pre-defined parameters. SVM was trained using the parameters previously mentioned.
6. Classification: quantification of the classification performance over 1625 samples using the SVM model trained in point 5.
7. Computing the total representation time. This is the sum of the results from points 2 and 4.
8. Computing of the total offline phase. It is defined as the sum of the results from points 3 and 5.
9. Computing of the total online phase, which consists of the sum of the results from points 2, 4, and 6.

**Table 7:** Comparison of time performance results.

Process No.	BOF-BoVW 3 Layers		BOF-BoVW 20 Layers		SIFT-BoVW	
	CPU (s)	Real (s)	CPU (s)	Real (s)	CPU (s)	Real (s)
1	0.31	0.30	0.46	0.39	0.32	0.26
2	294.87	295.68	392.12	353.83	486.92	292.70
3	73.00	18.39	9710.73	2871.72	3962.71	1354.93
4	107.59	27.93	148.17	38.52	533.62	137.86
5	93.78	94.37	100.78	100.69	16.23	16.33
6	27.81	27.97	31.17	31.36	6.20	6.20
7	402.46	323.62	540.30	392.35	1020.54	430.55
8	166.78	112.76	9811.51	2972.42	3978.94	1371.25
9	430.27	351.59	571.47	423.71	1026.74	436.75

In order to better understand the comparison of BOF-BoVW and SIFT-BoVW, we present the percentage increases for the listed cases Table 8. Increases are computed using the equation $I=(\left(V_{f}-V_{o}\right)/V_{o})\times 100$, where *I* is the percentage increase, $V_{f}$ the final value, and $V_{o}$ the initial value. *Increase B-S 3* means the percentage increase using BOF-BoVW with 3 layers as the initial value and SIFT-BoVW as the final value. The same reasoning is followed for *Increase B-S 20*, but relying on a BOF-BoVW with 20 layers as the initial value.

In terms of memory usage, the results for the sequence 01 train split of the Microsoft 7-Scenes dataset are shown in Table 9. The most relevant result can be observed in the first row, where SIFT descriptors need 1.9 GB. However, BOF descriptors with three layers need 49.4 MB, which translates into an increase of 3746% of storage needed. Using our heaviest 20 layers BOF representation leads to an increase of 593%. Maintaining descriptors over time is important if an implementation in a SLAM system is sought, due to the importance of reusing information from previous frames already visited, in order to speed up tasks such as loop closure detection. We observed that the BOW TF-IDF representations in both descriptors is almost identical, which can be explained by the fact that the model mainly depends on the codebook and the numbers of words in it. The other files that need to be stored are the codebook and model trained, and these remain in the megabyte scale in both cases.

### 4.5. Results of Loop Detection

**Figure 10:**
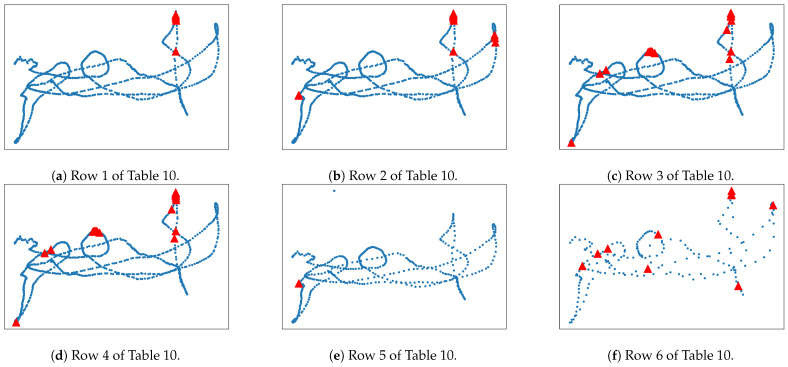
The detection of approved loop-closing candidates are shown in red for chess sequence 03. Each image corresponds to one row in Table 10, from left to right.

In Table 10, we display the results of the loop closure implementation. If we modify the parameter corresponding to the temporary consistency check ($\tau_{TC}$, TC req, TC), the approved rate is doubled, as shown in Figure 10b, which is contrasted with what is displayed in Figure 10a. The change in thresholds $\alpha^{+}$ and $\alpha^{-}$ does not have a significant impact on the discarded rate parameter, and just seven loops more are approved in Figure 10b,c.

Finally, we can also augment the gap between keyframes, which leads to a gain in processing speed, at the cost of reduced resolution. The lack of candidates and approved frames in Figure 10e is explained by the fact that we set the parameter keyframe to every two normal frames instead of one, and we did not adjust the remaining parameters to stay proportional with this new distribution of keyframes. This is displayed in Figure 10f. Despite having a lower value for the keyframes parameter, we achieved similar rates of approval and discard by means of tuning the relevant parameters.

The manner in which we implemented the loop-closing detection procedures is derived from counting with a bag of visual words representation for the scene classification phase. However, the fern approach in [30] seems to be adaptable to our descriptor in the following way: each BOF descriptor has 180 entries, so we can set 180 thresholds $\tau_{i}$ uniformly sampled and create a new binary vector, which contains a one if the corresponding BOF entry passes the threshold and zero otherwise.

In order to merge the results obtained in both parts, the classification and loop detection stages, a dataset needs to meet two requirements: to be divided in scenes and to contain a path that passes by those scenes. In this way, a semantic verification step immediately before the time consistency stage can be implemented in order to use this semantic information.

## 5. Conclusions

Scene recognition and classification are open problems in the robotics, vision, and pattern recognition fields. In this paper, we described a novel method able to cope with complex scenes at the time that keeps computational complexity low. Our method achieves performance comparable to that of more demanding architectures. The recognition and classification model we developed achieves performance that is comparable to that of other relevant models in a time with a significantly lower computing demand.

The main purpose of the BOF descriptors is to be lightweight, that is, to reduce computational complexity in both space (memory use and hardware resources) and processing time. Using a relatively shallow architecture of only three layers and configuring the online processes (descriptor extraction, BoW representation, and classification) took 596 s less than the one with SIFT and was 2.38× faster, which is an important result because of the calculations that the onboard machine of the robot must complete. Furthermore, the offline processes (codebook generation and model training) also are more than 20 times faster in CPU time with the three-layer configuration. This opens the possibility of considering the implementation of a training phase on board to adjust the models trained offline.

The best scene recognition results were achieved with a configuration of 20 layers per frame. The results are comparable to those obtained with SIFT-based models, at least on the the two datasets we considered here. Also, we implemented an efficient completed loop-closing module. Furthermore, our method was able to rely on semantic information derived from the scenes. A particularly relevant next step in our research is the implementation of this module in a lightweight semantic SLAM system.

We presented the results of our approach in several tables and figures in Section 4, which are comparable to those obtained by more popular methods. At the same time, the significantly less computation needed by our approach was proven in the corresponding analyses. We consider this latter attribute to be one of the main contributions of our work.

An additional advantage of our method is that the number of descriptors and their size take up less space in the CPU’s RAM. While SIFT-BoVW uses 1.9 GB, BOF-BoVW (20 layers) requires only 274 MB (Table 9). On some small-form-factor computers, it would be challenging to load the operating system and run the algorithm with SIFT; however, using the BOF descriptor for scene classification overcomes this issue. Remember that the longer the autonomous navigation journey, the more descriptors are needed for both SIFT and BOF.

### Future Work

A natural follow-up experiment involves testing the entire SLAM algorithm on the two datasets descxribed in this paper. Moreover, our model can be embedded in a robot with omnidirectional wheels to confirm that the point cloud capture remains unaffected by potential camera warping. Given the robot’s primarily smooth horizontal movement and the camera’s fixed position, the point cloud is anticipated to maintain a consistent distance from the floor to the sensor without any tilt.

Currently, classification methods using deep learning are very competitive tools and reach extensive generalization ranges. So, we will seek to move away from classification using SVM and opt for a deep learning model that classifies the BOFs of each layer of each frame of each scene. Unlike the images to be classified with these algorithms, in this method, the vectors are made up of 180 values. This enables reductions in the number of inputs in convolutional networks and in the number of parameters.

As a possible extension of our work, a different alternative is to consider descriptors other than BOF in order to consider the placement and sequence of each point in the depth matrix. This aims to bypass the projection of points onto the layers.

## Figures and Tables

**Table 1:** Datasets used.

Characteristic	Microsoft 7-Scenes	SUN RGB-D	OBTP
Year	2013	2015	2023
Camera	Kinect RGB-D	Intel RealSense, Asus Xtion, and Kinect v1/2 [21]	RealSense D455
Sensor type	Infrared camera and IR projector	Structured light and TOF	Active IR stereo
Depth resolution	640 × 480	628 × 468, 640 × 480, 640 × 480, and 512 × 424	640 × 480
Color resolution	640 × 480	1920 × 1080, 640 × 480, 640 × 48, and 1920 × 1080	640 × 480
Number of scenes	7	19	7
Number of images per scene	500 to 1000	80 to 600	200 to 800
Frame file formatting	Color (PNG), depth (PNG) image, and pose (txt)	RGB-D, depth, and segmentation maps	Color (PNG) and depth (PNG) image

**Table 2:** Classification results for BOF-BoVW and SIFT-BoVW using hold-out method with 25% test data.

BOF-BoVW	Precision	Recall	F1 Score	Support	SIFT-BoVW	Precision	Recall	F1 Score	Support
chess	0.99	0.98	0.99	263	chess	1.00	1.00	1.00	263
fire	0.99	1.00	0.99	239	fire	1.00	1.00	1.00	239
heads	0.98	1.00	0.99	247	heads	1.00	1.00	1.00	247
office	0.99	0.98	0.99	262	office	1.00	1.00	1.00	262
pumpkin	1.00	0.99	0.99	249	pumpkin	1.00	1.00	1.00	249
redkitchen	1.00	0.99	0.99	245	redkitchen	1.00	1.00	1.00	245
stairs	1.00	1.00	1.00	120	stairs	1.00	1.00	1.00	120
accuracy			0.99	1625	accuracy			1.00	1625
macro avg	0.99	0.99	0.99	1625	macro avg	1.00	1.00	1.00	1625
weighted avg	0.99	0.99	0.99	1625	weighted avg	1.00	1.00	1.00	1625

**Table 3:** Classification report for BOF-BoVW and SIFT-BoVW, using a different sequence as test data. Values rounded to 2 decimal places.

BOF-BoVW	Precision	Recall	F1 Score	Support	SIFT-BoVW	Precision	Recall	F1 Score	Support
chess	0.27	0.38	0.32	1000	chess	0.95	0.87	0.91	1000
fire	0.36	0.29	0.32	1000	fire	0.94	0.99	0.97	1000
heads	0.46	0.61	0.52	1000	heads	0.59	0.93	0.72	1000
office	0.28	0.45	0.34	1000	office	0.86	0.92	0.89	1000
pumpkin	0.56	0.37	0.45	1000	pumpkin	0.99	0.98	0.99	1000
redkitchen	0.15	0.09	0.11	1000	redkitchen	0.94	0.84	0.89	1000
stairs	0.05	0.01	0.01	500	stairs	0.09	0.00	0.00	500
accuracy			0.34	6500	accuracy			0.85	6500
macro avg	0.30	0.31	0.30	6500	macro avg	0.77	0.79	0.77	6500
weighted avg	0.32	0.34	0.32	6500	weighted avg	0.82	0.85	0.82	6500

**Table 4:** Classification report for the SUN RGB-D dataset rounded to two decimals. The test split contains 5050 frames.

Scene	Precision	Recall	F1 Score	Support
(**a**) BOF-BoVW				
study space	0.00	0.00	0.00	127
rest space	0.16	0.32	0.22	533
office	0.14	0.25	0.18	540
living room	0.14	0.07	0.10	255
library	0.35	0.03	0.05	221
lecture theatre	0.00	0.00	0.00	43
lab	0.00	0.00	0.00	223
kitchen	0.22	0.08	0.11	276
home office	0.00	0.00	0.00	128
furniture store	0.31	0.55	0.39	380
discussion area	0.00	0.00	0.00	117
dining room	0.00	0.00	0.00	96
dining area	0.15	0.04	0.06	237
corridor	0.29	0.16	0.20	196
conference room	0.00	0.00	0.00	207
computer room	0.29	0.03	0.05	67
classroom	0.19	0.25	0.21	520
bedroom	0.24	0.40	0.30	578
bathroom	0.30	0.23	0.26	306
accuracy			0.21	5050
macro avg	0.15	0.13	0.11	5050
weighted avg	0.18	0.21	0.17	5050
**Scene**	**Precision**	**Recall**	**F1 Score**	**Support**
(**b**) SIFT-BoVW				
study space	0.02	0.01	0.01	127
rest space	0.15	0.20	0.17	533
office	0.22	0.34	0.27	540
living room	0.10	0.11	0.10	255
library	0.14	0.08	0.10	221
lecture theatre	0.02	0.05	0.03	43
lab	0.09	0.00	0.01	223
kitchen	0.14	0.15	0.15	276
home office	0.11	0.12	0.12	128
furniture store	0.36	0.54	0.43	380
discussion area	0.02	0.02	0.02	117
dining room	0.07	0.07	0.07	96
dining area	0.11	0.08	0.09	237
corridor	0.10	0.10	0.10	196
conference room	0.18	0.08	0.11	207
computer room	0.10	0.15	0.12	67
classroom	0.33	0.23	0.27	520
bedroom	0.29	0.26	0.27	578
bathroom	0.36	0.35	0.36	306
accuracy			0.21	5050
macro avg	0.15	0.15	0.15	5050
weighted avg	0.20	0.21	0.20	5050

**Table 5:** Accuracy comparison of descriptors tested on the SUN RGB-D dataset. In this case, the values are truncated. The GIST results were extracted from [20].

	BOF-BoVW	SIFT-BoVW	GIST RGB	GIST DEPTH	GIST RGB + DEPTH
Accuracy	20.53	20.87	19.7	20.1	23

**Table 6:** Classification report for the OBPT dataset rounded to two decimals.

Scene	Precision	Recall	F1 Score	Support
(**a**) 7 scenes’ classification				
office_1	0.89	0.89	0.89	135
office_2	0.95	0.53	0.68	135
laboratory	0.63	0.74	0.68	135
corridor_1	0.53	0.35	0.43	110
corridor_2	0.55	0.77	0.64	135
corridor_3	0.70	0.77	0.73	135
bathrooms	0.66	0.70	0.68	135
accuracy	0.69	0.69	0.69	920
macro avg	0.70	0.68	0.68	920
weighted avg	0.71	0.69	0.68	920
(**b**) 4 scenes’ classification				
office	0.91	0.87	0.89	165
laboratory	0.81	0.80	0.80	165
corridor	0.94	0.98	0.96	165
bathrooms	0.78	0.79	0.79	165
accuracy	0.86	0.86	0.86	660
macro avg	0.86	0.86	0.86	660
weighted avg	0.86	0.86	0.86	660

**Table 8:** Percent increases in time consumption.

Process No.	Increase B-S 3		Increase B-S 20	
	CPU (%)	Real (%)	CPU (%)	Real (%)
1	1	−13	−31	−33
2	65	−1	24	−17
3	5328	7266	−59	−53
4	396	394	260	258
5	−83	−83	−84	−84
6	−78	−78	−80	−80
7	154	33	89	10
8	2286	1116	−59	−54
9	139	24	80	3

**Table 9:** Comparison of storage usage.

File	BOF-BoVW 3 Layers	BOF-BoVW 20 Layers	SIFT-BoVW
Raw descriptors	49.4 MB	274 MB	1.9 GB
BoVW TF-IDF representation	19.9 MB	20 MB	20 MB
Codebook	1.47 MB	1.47 MB	524 KB
Trained model	32.9 MB	35.4 MB	15.8 MB

**Table 10:** Loop closure detection results for the chess sequence 03.

$\alpha^{+}$	$\alpha^{-}$	*N*	τ ${}_{N}$	TC Req	TC	τ ${}_{\mathrm{TC}}$	Key Frames	Candidates	Approved	Discarded
0.6	0.15	−31	0.1	5	31	60	1000	34	7	0
0.6	0.15	−30	0.1	3	15	60	1000	35	16	0
0.6	0.15	−15	0.05	3	15	60	1000	70	19	17
0.5	0.3	−15	0.05	3	15	60	1000	73	26	17
0.5	0.3	−15	0.05	3	15	60	500	8	1	9
0.5	0.3	−2	0.05	1	3	20	200	24	10	11

## Data Availability

The code and pretrained models can be found at https://github.com/victorlomas/public (accessed on 2 October 2023).

## Abbreviations

The following abbreviations are used in this manuscript:

BOF	Boundary object function
BoVW	Bag of visual words
ORB	Oriented FAST and rotated BRIEF
RBF	Radial basis function
SIFT	Scale-invariant feature transform
SLAM	Simultaneous localization and mapping
SURF	Sped-up robust features
SVM	Support vector machine
Tf-idf	Term frequency-inverse document frequency
